# Peedanil Gold, Herbo-Mineral Formulation, Moderates Cytokine Levels and Attenuates Pathophysiology in Monosodium Iodoacetate Induced Osteoarthritis in SD Rat Model

**DOI:** 10.3389/fphar.2022.883475

**Published:** 2022-05-04

**Authors:** Acharya Balkrishna, Sandeep Sinha, Shadrak Karumuri, Jyotish Srivastava, Swati Haldar, Anurag Varshney

**Affiliations:** ^1^ Drug Discovery and Development Division, Patanjali Research Institute, Haridwar, India; ^2^ Department of Allied and Applied Sciences, University of Patanjali, Haridwar, India; ^3^ Department of Biology, Patanjali Research Institute, Haridwar, India; ^4^ Department of Chemistry, Patanjali Research Institute, Haridwar, India; ^5^ Department of Microbiology, Patanjali Research Institute, Haridwar, India; ^6^ Special Centre for Systems Medicine, Jawaharlal Nehru University, New Delhi, India

**Keywords:** Peedanil Gold, MIA, osteoarthritis, Kellgren & Lawrence, hyperalgesia, allodynia

## Abstract

The inflammatory cartilaginous degeneration of the articular joints, mostly those of knee, hips and hands, is osteoarthritis (OA). The available treatment strategies for osteoarthritis are designed for pain relief, molecular targeting, cartilage regeneration and surgical intervention. However, meta-analysis of clinical trials has shown these strategies to be sub-optimal, thereby, eliciting a need for investigating alternative options. The herbo-mineral formulation, Peedanil Gold (PN-G) has been used against joint pains and inflammation. In the current study, anti-osteoarthritic effects of PN-G were investigated in rat model of OA, induced by intra-articular injection of monosodium-iodoacetate. PN-G treatment improved the clinical and Kellgren & Lawrence scores; and rescued the osteoarthritic rats from hyperalgesia and allodynia. Besides, PN-G treatment ameliorated joint inflammation and abrogated *in vivo* osteoarthritic pathology through effective cartilage regeneration, measured radiologically and histopathologically. PN-G also reduced the levels of interleukin-6 (IL-6) and interleukin-1 beta (IL-1β), in a dose dependent manner, in inflamed human macrophagic THP-1 cells, thereby, reaffirming its anti-inflammatory property at cytosafe concentrations. Ultra High performance liquid chromatography (UHPLC) revealed the presence of several analgesic and anti-inflammatory phytocompounds, like ellagic acid, guggulsterone E, guggulsterone Z, 5-(hydroxymethyl) furfural, corilagin, cinnamic acid, ferulic acid, gallic acid and protocatechuic acid in PN-G. In conclusion, this study has succinctly demonstrated that PN-G is capable of relieving the clinical symptoms of osteoarthritis, which is measurable through the established osteoarthritic serum biomarker, Cartilage Oligomeric Matrix Protein (COMP).

## 1 Introduction

Osteoarthritis, the “wear and tear” of the joints, can practically affect any articular joint in the body, although, a distinct preference for those of knees, hips and hands has been observed.^1^ Studies, so far have failed to provide a consensus on the association between osteoarthritis and mortality (Kloppenburg and Berenbaum, 2020). However, the ever-increasing social and economic burden of osteoarthritis due to disability-adjusted life-years (DALYs), because of overall increase in human life-span has reared up as a big challenge. The gravity of this unmet medical need is reflected in the fact that Osteoarthritis Research Society International (OARSI), has described osteoarthritis as a “Serious Disease” in its white paper submitted to US-FDA.^2^

Osteoarthritis management includes conventional treatments focused on pain relief, advanced strategies targeting molecular pathways involved in the disease pathogenesis, regenerative approach and surgical treatment. Meta-analysis of the outcomes of the clinical trials on all these osteoarthritic management approaches reveals that a full-proof treatment for this disease is still lacking (Kloppenburg and Berenbaum, 2020). The most commonly adopted osteoarthritic management strategies and treatments include symptomatic alleviation, like pain relief through analgesics (mostly paracetamol), non-steroidal anti-inflammatory drugs (NSAIDs) and steroidal injections. Despite limited proven efficacy (Leopoldino et al., 2019), paracetamol is widely used to treat osteoarthritis (Kloppenburg and Berenbaum, 2020). Meta-analysis of clinical trials on conventional NSAIDs, intra-articular hyaluronate and corticosteroids injections revealed that these trials were inconclusive (Gregori et al., 2018). The network meta-analysis of forty-seven randomized clinical trials with a total sample size of 22,037 knee osteoarthritic patients and a minimum of 12 months follow-up, demonstrated uncertainty of the effect size of pain moderation compared to the placebo (Gregori et al., 2018). In fact, Gregori et al. (2018) recommended larger randomized clinical trials to address the uncertainty on the efficacies of the prescribed medications for knee osteoarthritis (Gregori et al., 2018).

Meta-analyses of 754 trials on Cyclooxygenase-2 (COX-2) inhibitors and traditional NSAIDs determined the adjusted rate ratio for the major vascular events for treatment vs. placebo to be 1.41 (at 95% confidence limit with *p* = 0.0036), thereby, recognizing the association of these medications with increased cardiac risks (Coxib and traditional NSAID Trialists’ (CNT), 2013). Intra-articular corticosteroid injections might increase cartilage loss (McAlindon et al., 2017). Clinical trials on its alternative, intra-muscular injection are also far from providing clarity on the suitability of the approach (Kloppenburg and Berenbaum, 2020). Capsaicin, an agonist for the transient receptor potential cation channel subfamily V member 1 (TRPV1) is already being used as topical analgesic for osteoarthritis, although its injectable version, CNTX-4975 did not show promising anti-osteoarthritic activity in the clinical trial (Stevens et al., 2019). Other clinical trials on inhibitors for interleukin-1 alpha/beta (IL-1α/β) (Lutikizumab) (Kloppenburg et al., 2019), tumor necrosis factor (TNF) (Etanercept) (Kloppenburg et al., 2018) and calcitonin gene-related peptide (CGRP) (Galcanezumab) (Jin et al., 2018) did not offer encouraging outcomes, except for Etanercept that improved the radiologically detectable joint remodeling, but without any pain relief. Efficacy of regenerative medicine involving mesenchymal stem cell (Xing et al., 2018) or platelet rich plasma injection (Muchedzi and Roberts, 2018) is still in infancy. Surgical treatment of osteoarthritis is a short-term fixation of the situation which fails and requires reversal after a while, thereby, increasing the lifetime risk (Kloppenburg and Berenbaum, 2020). Thus, each of the available strategies for treating osteoarthritis is laced with demerits, eliciting the need for investigating alternative treatment options.

In this study, an Ayurvedic herbo-mineral medicine, Peedanil Gold (PN-G) was evaluated for potential anti-osteoarthritic effect in Monosodium iodoacetate (MIA) induced rat model of osteoarthritis. PN-G is an approved Ayurvedic medicine, manufactured by Divya Pharmacy, Haridwar, India (manufacturing license numbers: Uttra.Ayu-67/2005 and UK.AY-274/2013). Clinical dosage of PN-G has been recommended as two tablets (540 mg each), twice a day, with lukewarm water. PN-G is formulated with, a calcium rich mineral formulation, *Mukta Shukti Bhasma*, two metallic sub-formulations, *Mahavat Vidhvansan Ras* and *Vrihatvat chintamani Ras*, two poly-herbo-mineral sub-formulations, *Punarnavadi Mandoor* and *Amvatari Ras*, along with a resinous exudate of *Commiphora wightii* (Guggul Shuddh), and inert tablet binding agents. The preparation of individual sub-formulations has been mentioned in classical texts of Ayurveda (Table 1). PN-G is recommended for joint pain and swelling, which has been the rationale behind designing this study to assess its potential anti-osteoarthritic properties; and to obtain insight into its mode-of-action.

**TABLE 1:** Composition of Peedanil Gold (PN-G).

Name of the component	Identity of the component	Form used	Quantity (mg)	Classical text references	Page nos.
Classical components					
Punarnavadi Mandoor	Classical formulation	Powder	60	Ayurved Sarsangrah, edition 2010	499
Guggul Shuddh	Exudate of *Commiphora wightii*	Resin	250	Bhav Prakash Nighantu, edition 2006	205
Mukta Shukti Bhasma	Classical formulation	Powder	60	Ras Tarangini, edition 2004	295–298
Mahavat Vidhvansan Ras	Classical formulation	Powder	60	Ayurved Sarsangrah, edition 2010	373
Amvatari Ras	Classical formulation	Powder	60	Ayurved Sarsangrah, dition 2010	260
Vrihatvat Chintamani Ras	Classical formulation	Powder	10	Bhaishajya Ratnavali, eighteenth edition	543–544
Excipients					
Gum acacia	Exudate of *Acacia arabica*	Resin	8	Indian Pharmacopoeia 2014, Vol III	3172
Talcum	Hydrated magnesium silicate	Powder	8	Indian Pharmacopoeia 2014, Vol III	2821
MCC	Microcrystalline cellulose	Powder	16	Indian Pharmacopoeia 2014, Vol III	2229
Croscarmellose sodium	Sodium carboxymethyl cellulose	Powder	8	Indian Pharmacopoeia 2014, Vol III	1469–1470

## 2 Results

### 2.1 PN-G Is Phytochemically Enriched With Analgesic and Anti-Inflammatory Bioactive Compounds

**FIGURE 1:**
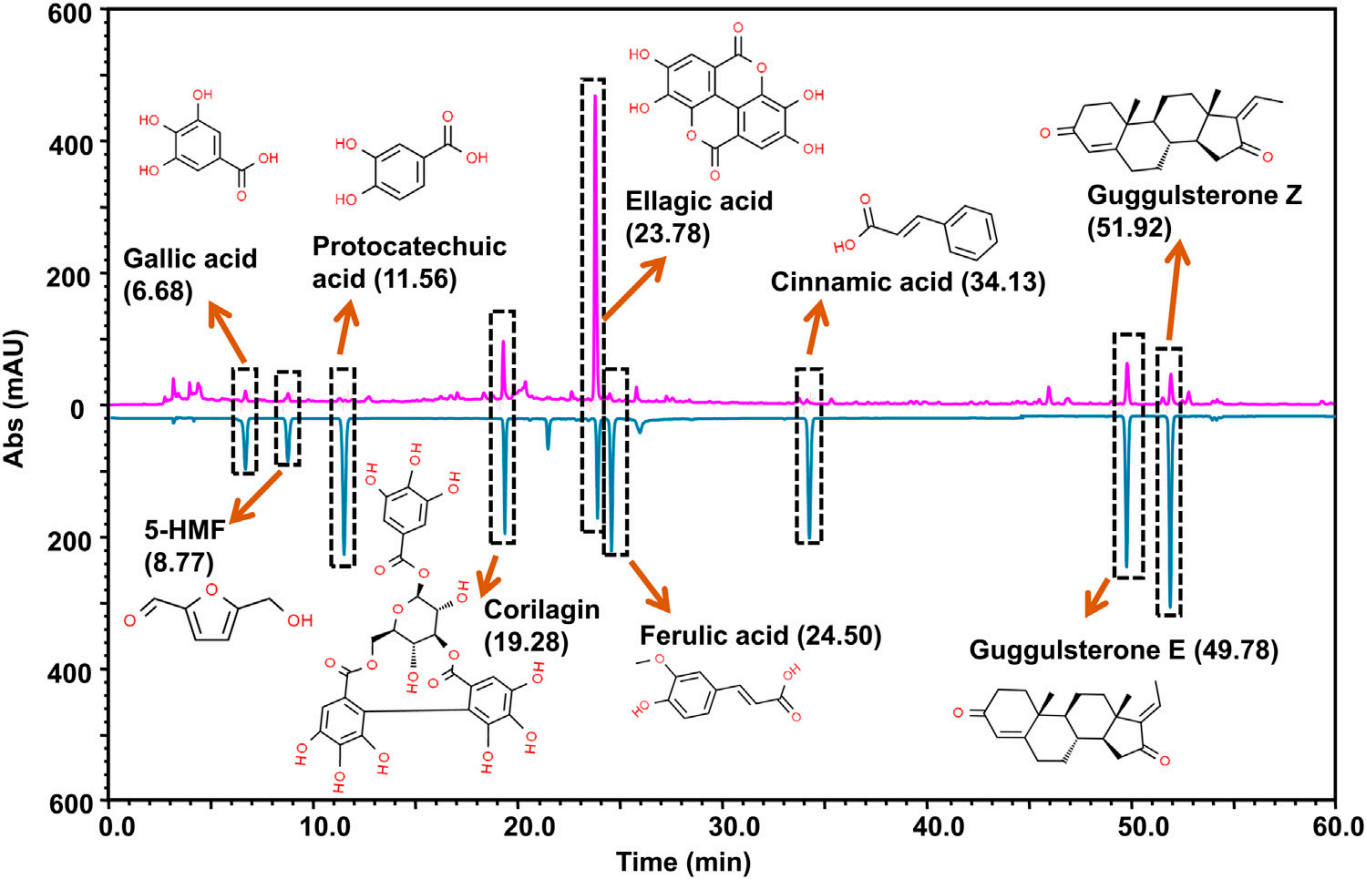
Marker compound analysis. PN-G powder (chromatogram shown in pink) was subjected to UHPLC-PDA analysis by employing reference standards (chromatogram shown in blue). The chromatograms were recorded at 250 nm. By correlating the chromatograms vis-à-vis the pure analytical standards, UHPLC analysis detected the presence of nine marker phytocompounds in a 540 mg PN-G tablet, namely, gallic acid (7.5 µg), 5-(hydroxymethyl)furfural (45.5 µg), protocatechuic acid (6.0 µg), corilagin (21.5 µg), ellagic acid (1144.0 µg), ferulic acid (8.5 µg), cinnamic acid (11.0 µg), guggulsterone E (81.0 µg) and guggulsterone Z (70.0 µg). The chemical structures of the respective compounds are provided with the chromatogram.

The wide-spread recommendation of PN-G as a pain-relieving agent in the classical Ayurvedic texts, encouraged us to conduct a detailed compositional scrutiny of its phytochemicals. The Ultra High Performance Liquid Chromatography (UHPLC) using a Photodiode Array (PDA) detector (UHPLC-PDA analysis) identified nine prominent phytocompounds, namely, ellagic acid, guggulsterone E, guggulsterone Z, 5-(hydroxymethyl) furfural, corilagin, cinnamic acid, ferulic acid, gallic acid and protocatechuic acid (Figure 1). Each 540 mg tablet of PN-G predominantly contained ellagic acid (1,144 µg), followed by guggulsterone E (81 µg) and Z (70 µg). 5-(hydroxymethyl) furfural at 45.5 µg/tablet is present at a concentration nearly half that of guggulsterone E, and corilagin (21.5 µg/tablet) at almost half that of 5-(hydroxymethyl) furfural. Cinnamic, ferulic, gallic, and protocatechuic acids were found at 11, 8.5, 7.5 and 6 µg/tablet. Interestingly, six out of nine identified phytoconstituents of PN-G, namely, ellagic acid (Naghizadeh et al., 2016; BenSaad et al., 2017), corilagin (Jin et al., 2013; Moreira et al., 2013), ferulic acid (Priebe et al., 2018; Zhang et al., 2018), gallic acid (Trevisan et al., 2014; BenSaad et al., 2017) and protocatechuic acid (Lende et al., 2011), have both anti-nociceptive and anti-inflammatory effects. The remaining phytoconstituents, guggulsterones (Shah et al., 2012; Zhang et al., 2016), 5-(hydroxymethyl) furfural (Kong et al., 2019) and cinnamic acid (Liao et al., 2012) are known for their anti-inflammatory properties. Pertinently, a recent study has reported the protective effect of ellagic acid against osteoarthritis; nociception and inflammation being its two major pathophysiologies (Lin et al., 2020). Taken together, the compositional analysis of PN-G has helped us in identifying the plausible reasons behind its popularity as a traditional treatment for joint pains. Subsequently, *in vitro* and *in vivo* validations of the implicated anti-osteoarthritic effect of PN-G were conducted.

### 2.2 PN-G Exerts Its Anti-Inflammatory Effect at Cytologically Safe Concentrations

**FIGURE 2:**
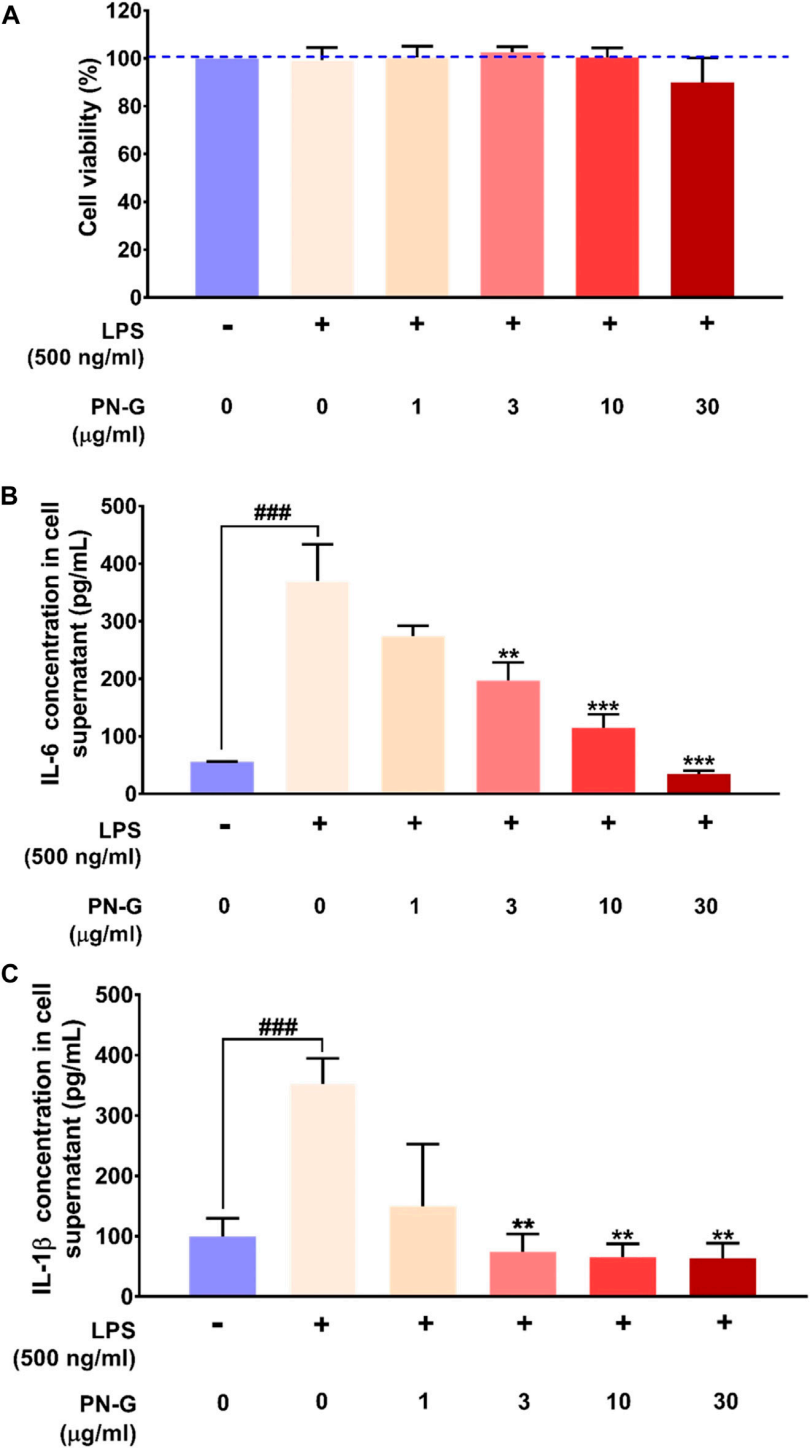
PN-G exhibited anti-inflammatory property at cytosafe doses. **(A)** Bar graph showing the *in vitro* safety of PN-G treatment on THP-1 cells at different concentrations and, by extension, the suitability of the LPS-induced *in vitro* inflammatory model for subsequent experiments. **(B,C)** Graphical representations of the effect of PN-G treatment on release of different pro-inflammatory cytokines, namely, IL-6 **(B)** and IL-1β **(C)** from LPS-induced inflamed THP-1 cells. Data is represented as mean ± SEM. The statistical significance of the observed effect was analyzed through one-way ANOVA followed by Dunnett’s multiple comparison test and represented as ^###^ for *p* < 0.001, when compared with normal cells, untreated with LPS or PN-G. When the comparison was with the PN-G untreated, LPS-induced cells, the statistical significance is depicted with ** and *** for *p* < 0.01 and <0.001, respectively.

Cytologically safe anti-inflammatory dose of PN-G was determined through alamar blue staining of LPS induced, PN-G-treated, human macrophagic THP-1 cells. Different doses of PN-G over a range of 1–30 μg/ml, were noted to be cytologically safe on the THP-1 cells. Besides, the LPS induction alone or in combination with PN-G treatment did not exhibit any adverse effects on the cells (Figure 2A). This confirmed the suitability of the *in vitro* inflammatory model for subsequent studies to evaluate the anti-inflammatory effect of PN-G by measuring the levels of secreted pro-inflammatory cytokines, like, interleukin (IL-6) and interleukin-1 beta (IL-1β) through ELISA. The THP-1 cells, when exposed to LPS, elicited an inflammatory response, as evident from significant increases in the secreted IL-6 and IL-1β levels, compared to the normal LPS-un-induced cells. Statistically significant decreases in the levels of both IL-6 and IL-1β, in comparison to the LPS-induced untreated cells, were observed in treatments with ≥3 μg/ml of PN-G (Figures 2B,C). PN-G treatment reduced the level of secreted IL-6 in a dose-dependent manner (Figure 2B). The effect on IL-1β was dose-dependent until 3 μg/ml of PN-G, that brought down its level to a limit, which was comparable with the normal cells. Higher concentrations of PN-G were found to be preventive against IL-1β secretion beyond the basal level (Figure 2C). From these observations, we concluded that PN-G, at cytological safe concentrations, could suppress *in vitro* inflammatory responses that involved pro-inflammatory cytokines, IL-6 and IL-1β.

### 2.3 Osteoarthritic clinical and Kellgren-Lawrence Scores Decreased With PN-G Treatment

**FIGURE 3:**
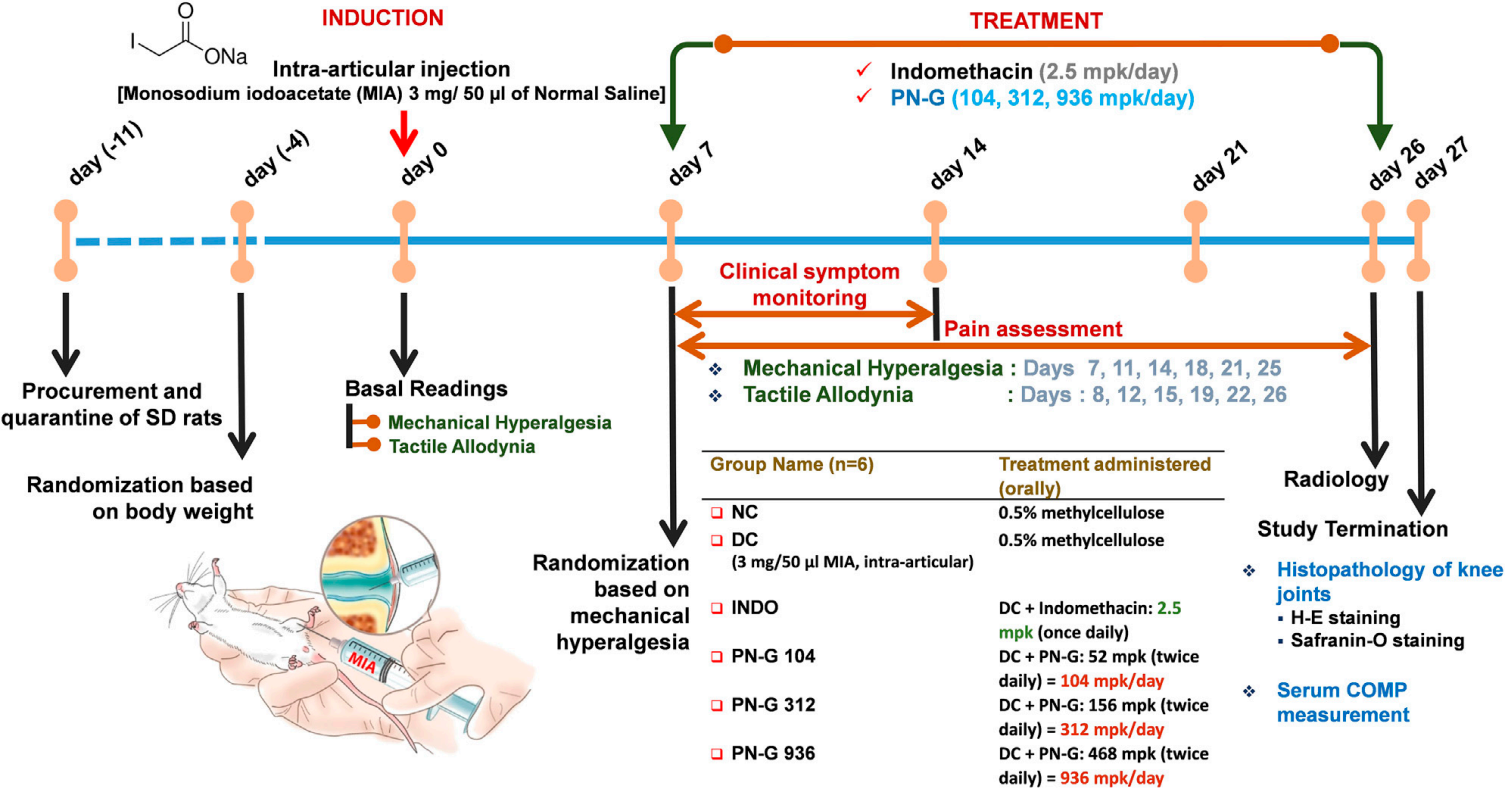
Schematic of the *in vivo* experiments. Following quarantine and acclimatization, baseline readings of the employed pain parameters were recorded for all animals. Thereafter, MIA was injected into the right knee joints of all animals except those in the NC. Rats in NC group were administered saline by intra-articular route. Seven days post-MIA injection, animals were assessed for the development of mechanical hyperalgesia for randomization to receive different designated treatments, thenceforth. Rats received either indomethacin (2.5 mpk/day) or three different doses of PN-G (104, 312 and 936 mpk/day). Animals in NC and DC groups received methylcellulose. Mechanical hyperalgesia and tactile allodynia were recorded at an interval of every 3–4 days until day 26, when the animals were additionally subjected to radiological examination. On day 27, animals were euthanized and blood was collected for measurement of the serum osteoarthritic biomarker, COMP. The right knee joints were harvested for histopathological evaluation through H-E and Safranin-O staining.

**FIGURE 4:**
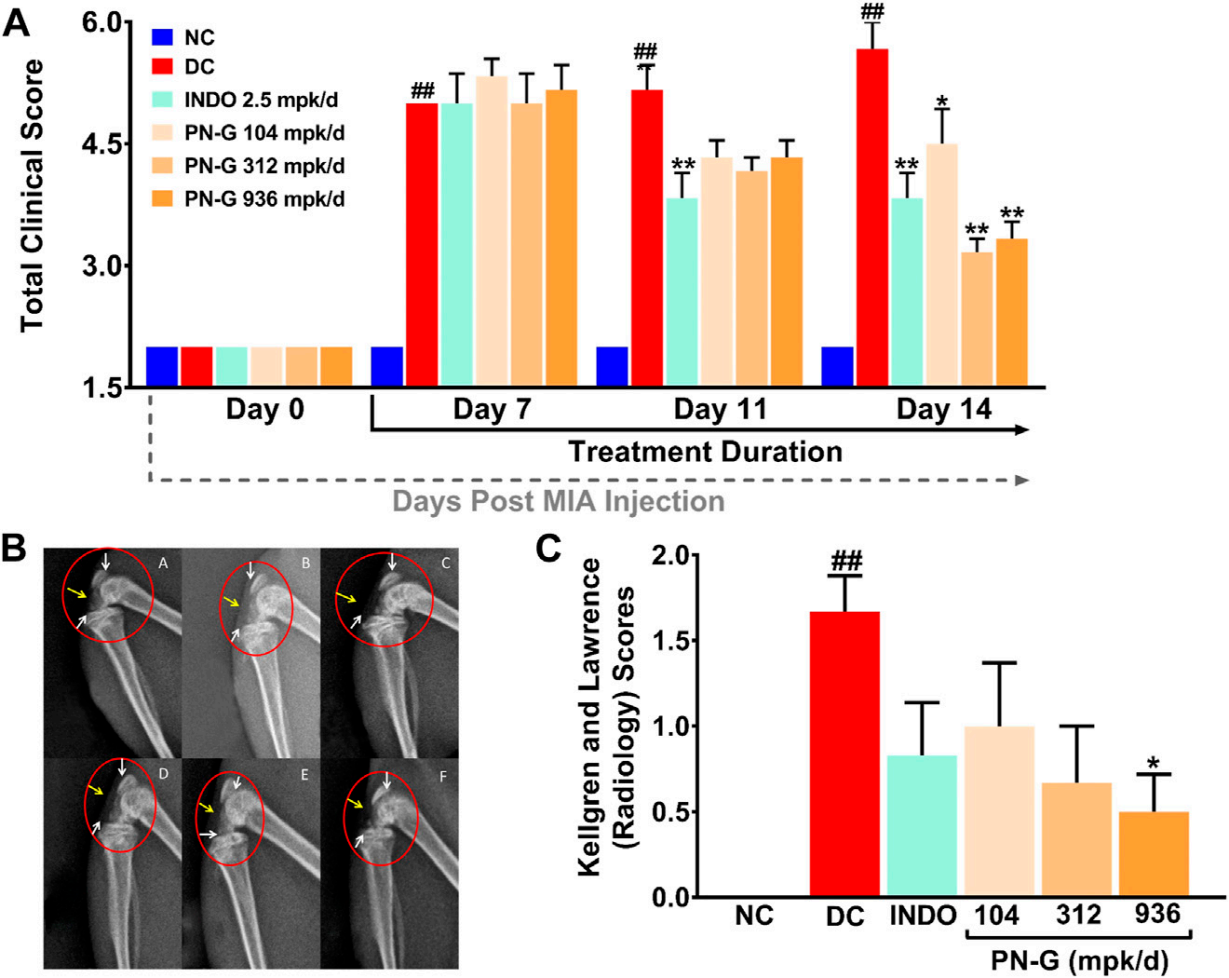
PN-G exhibited clinical abrogation of osteoarthritis. **(A)** Bar graph showing the effect of PN-G treatment on osteoarthritic clinical scores determined from joint swelling and limping. **(B)** Representative radiograms of right knee joints of rats belonging to different experimental groups, namely, NC **(A)**, DC **(B)**, INDO **(C)**, PN-G 104 **(D)**, PN-G 312 **(E)** and PN-G 936 **(F)** depicting various radiological features, like, joint space & radiodensity (yellow arrow) and surfaces of femoral condyles and proximal tibia (white arrow). **(C)** Bar graph showing the effect of PN-G treatment on the Kellgren and Lawrence scores, that depict the extent of osteoarthritic severity. Data is represented as mean ± SEM (*n* = 6 animals per group) and was statistically analyzed using two-way ANOVA followed by Tukey’s multiple comparison. ^##^ indicates significant difference with respect to NC (*p* < 0.01) whereas * and ** depict a statistically significant effect when compared to DC (*p < 0.05 and **p < 0.01).

Inflammation plays an important role in the pathogenesis of osteoarthritis (Chow and Chin, 2020), therefore, the ameliorative effect of PN-G on osteoarthritic pathology was evaluated *in vivo* using MIA-induced osteoarthritic model of rat (Figure 3). Symptomatically, osteoarthritis manifests itself as joint swelling and consequent gait alterations, observed as limping. Therefore, osteoarthritic pathology and effect of any treatment thereof was clinically scored. Such scoring over 14 days, verified the establishment of the osteoarthritic model through significantly increased tallies, counting till 5 in the DC group after 7 days of MIA injection, compared to NC. The clinical scores in rest of the experimental groups hovered around that of DC. Within a week of indomethacin and PN-G treatments, the clinical scores exhibited a decline. The observed decrease in clinical score was statistically significant in the indomethacin-treated INDO group from day 11 itself, with a decline from 5.00 ± 0.37 on day 7 to 3.83 ± 0.31 on day 11 (*p* < 0.01), without further decrease by day 14. The clinical scores of PN-G treated groups on day 11 visibly floated around the INDO group, despite, being statistically insignificant compared to the DC; however, by day 14, these scores were significantly reduced relative to the DC group. With PN-G treatment at 104 mpk/d, the clinical score reduced by ∼ 20.63% relative to the DC group. The clinical score decreased further with (∼44.10%) treatment of PN-G. However, PN-G treatment at 936 mpk/d, showed a similar decrease (∼41.27%) in the clinical scores as that of 312 mpk/d PN-G treated group (Figure 4A). As expected the representative radiogram of healthy knee joint belonging to the NC group was without distension showing usual radiodensity, regular joint spacing (Figure 4BA, yellow arrow) and normal surfaces of femoral condyles and proximal tibia (Figure 4BA, white arrow). The radiogram pertaining to the DC group showed distended joint with reduced spacing (yellow arrow), increased radiodensity and roughened femoral condyle and proximal tibia surface indicating new osteophyte formation (white arrow) (Figure 4BB). The representative radiogram from the INDO group receiving treatment with indomethacin, sported a knee with regular joint spacing, usual radiodensity without any distension (yellow arrow). Osteophytic reactivity was reduced on the femoral condyles and proximal tibia, displaying near normal surfaces (white arrow) (Figure 4BC). The characteristic osteoarthritic radiographic features showed amelioration with PN-G treatments (Figure 4BD–F). Additionally, the extent of the osteoarthritic pathology was evaluated through Kellgren and Lawrence (K & L) scoring of the radiograms (Figure 4C) (Kellgren and Lawrence, 1957; Belkhodja et al., 2017; Abdel Jaleel et al., 2020). These scores corroborated with the inferences drawn from earlier clinical scoring and showed that the DC group had an average K & L score of 1.67 ± 0.21 against a 0 score for NC group. While this score decreased with indomethacin (0.83 ± 0.31) and PN-G treatments (1.00 ± 0.37 for 104 mpk/d, 0.67 ± 0.33 for 312 mpk/d and 0.50 ± 0.22 for 936 mpk/d), its statistically significant lessening, compared to DC group, was observed only in case of 936 mpk/d dose of PN-G. Taken together, these observations proved that PN-G treatment indeed promoted clinical reversal of osteoarthritic symptoms and macroscopic pathologies.

### 2.4 PN-G Treatment Relieved the Osteoarthritic Rats From Mechanical Hyperalgesia and Tactile Allodynia

**FIGURE 5:**
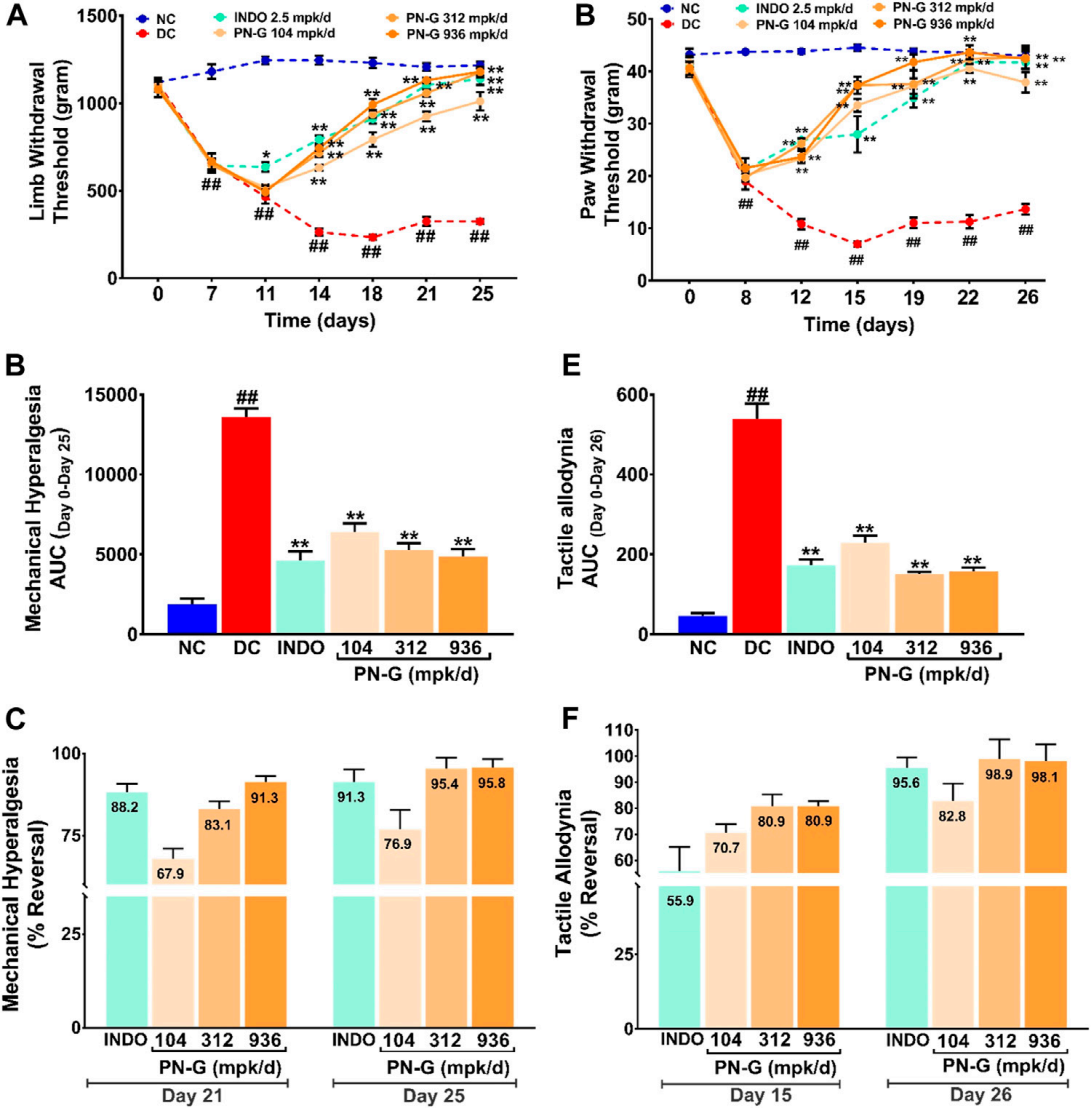
PN-G exhibits dose-dependent reversal of MIA-induced mechanical hyperalgesia and tactile allodynia. **(A,D)** Line graphs showing the effects of PN-G treatments on MIA-induced mechanical hyperalgesia **(A)** and tactile allodynia **(D)** spanning over the entire experimental regime of 26 days. Data was analyzed through two-way ANOVA followed by Tukey’s multiple comparison. ^##^ indicates significant difference with respect to NC (*p* < 0.01) whereas * and ** depict a statistically significant effect when compared to DC (*p < 0.05 and **p < 0.01). **(B,E)** Quantitative representations of the effects of PN-G treatments on mechanical hyperalgesia **(B)** and tactile allodynia **(E)** as area under the curves (AUCs) calculated from data shown in **(A)** and **(D)**. The data was statistically analyzed by employing one-way ANOVA followed by Dunnett’s multiple comparison test. ^##^ designates significant difference with respect to NC (*p* < 0.01) whereas ** depicts a statistically significant effect when compared to DC (*p* < 0.01). **(C,F)** Bar graphs showing the comparisons between the inhibitory effects, seen on mechanical hyperalgesia **(C)** and tactile allodynia **(F)**, due to indomethacin (INDO) and PN-G treatments. Values within the bars represent percentage reversal. All data was represented as mean ± SEM (*n* = 6 animals per group).

Joint swelling and inflammation associated with osteoarthritis lead to pain sensation (Eitner et al., 2017). Therefore, the effect of PN-G treatments on alleviation of the pain sensation arising from mechanical hyperalgesia and tactile allodynia was evaluated through Pressure Application Mechanics (PAM) and Von Frey tests, respectively. While a healthy SD rat could bear a weight between 1,000 and 1,250 g, the average weight tolerance gradually decreased with induction of osteoarthritis until the limb withdrawal threshold (LWT) reached around 600 g by a week after disease induction. The LWTs in all the group, except INDO, continued to decline to 500 g until day 11. The LWT of INDO group was steady until day 11. All the treatment groups experienced increase in their LWTs, albeit at different paces, from day 11 onwards, with INDO group showing a faster rise. Nevertheless, the increase in the LWTs of 312 and 936 mpk/d doses of PN-G caught up with that of INDO by day 14. The LWT of the group treated with 104 mpk/d of PN-G, rose more gradually than the rest. The LWT of the DC group declined to 250 g by day 14, beyond which, no further drop was observed (Figure 5A). The areas under the curves (AUCs) plotted from the changes in the LWTs over 25 days, from different groups, provided a quantitative visualization of the modulation of this pain assessment. DC group with ever increasing mechanical hyperalgesia showed maximum value for AUC (13,601.70 ± 533.70). The AUCs of the INDO and PN-G 312 and 936 groups were reduced to one-third of the DC group. The decrease in the AUC of PN-G 104 group to half that of DC, was slightly less compared to the other treatment groups (Figure 5B). The efficiencies of different treatments in relieving mechanical hyperalgesia were compared as % reversal (Figure 5C). By day 21, PN-G treatments of 312 and 936 mpk/d were nearly as effective as the positive control indomethacin in reversing mechanical hyperalgesia (88.2% in INDO vs. 83.1 and 91.3% in PN-G 312 and 936, respectively). However, PN-G treatment at 104 mpk/d was less efficient than indomethacin with 67.9% reversal. A similar trend was reflected on day 25 as well. These observations confirmed that osteoarthritis associated hyperalgesia is effectively relieved with PN-G treatments.

A similar outcome was observed in case of Von Frey test that measured tactile allodynia. It was found that induction of osteoarthritis with MIA increased the pain sensitization to an otherwise innocuous stimulus. The pain response was measured as the pressure at which the animals withdrew their paws from the source of experimental pressure (PWT). Higher PWT indicated alleviation of allodynia whereas, decrease in PWTs suggested increased allodynia. The PWT in DC group continued to decrease from 40 g on day 0 to below 20 g on day 8 until day 12, when a new threshold of 10 g was reached. With commencement of treatments on day 7, an almost immediate halt on the decline in PWTs was noticed on day 8. Reversal of allodynia, noted as increase in PWTs, was found to be comparable for all the treatment groups until day 12, beyond which PN-G treated groups showed steeper rise in their PWTs as compared to the positive control INDO group. PN-G treatment at 936 mpk/d in fact showed a PWT comparable to that of NC by day 19, thus, indicating almost complete alleviation of allodynia. These observations revealed that PN-G treatment, like indomethacin, could promptly cease the progression of allodynia, which otherwise exacerbated considerably in the absence of treatments (Figure 5D). A quantitative visualization of the treatment effects on allodynia through AUCs showed that, like mechanical hyperalgesia, allodynia was also reduced by two-third in case of indomethacin and PN-G 312 and 936 mpk/d treatments. PN-G treatment with 104 mpk/d could reduce allodynia by a little more than half of DC (Figure 5E). A comparison of the pain relieving efficacies between indomethacin and PN-G on day 15, showed latter to be more effective at all doses. However, by day 26, indomethacin and 312 and 936 mpk/d doses of PN-G were comparably effective in alleviating allodynia, whereas, 104 mpk/d PN-G was slightly less potent in this regard (Figure 5F). Taken together, an overall analgesic property of PN-G has been established through these observations from pain behavior assessments.

### 2.5 PN-G Treatment Ameliorated Joint Inflammation and Abrogated *In Vivo* Osteoarthritic Pathology Through Effective Cartilage Regeneration

**FIGURE 6:**
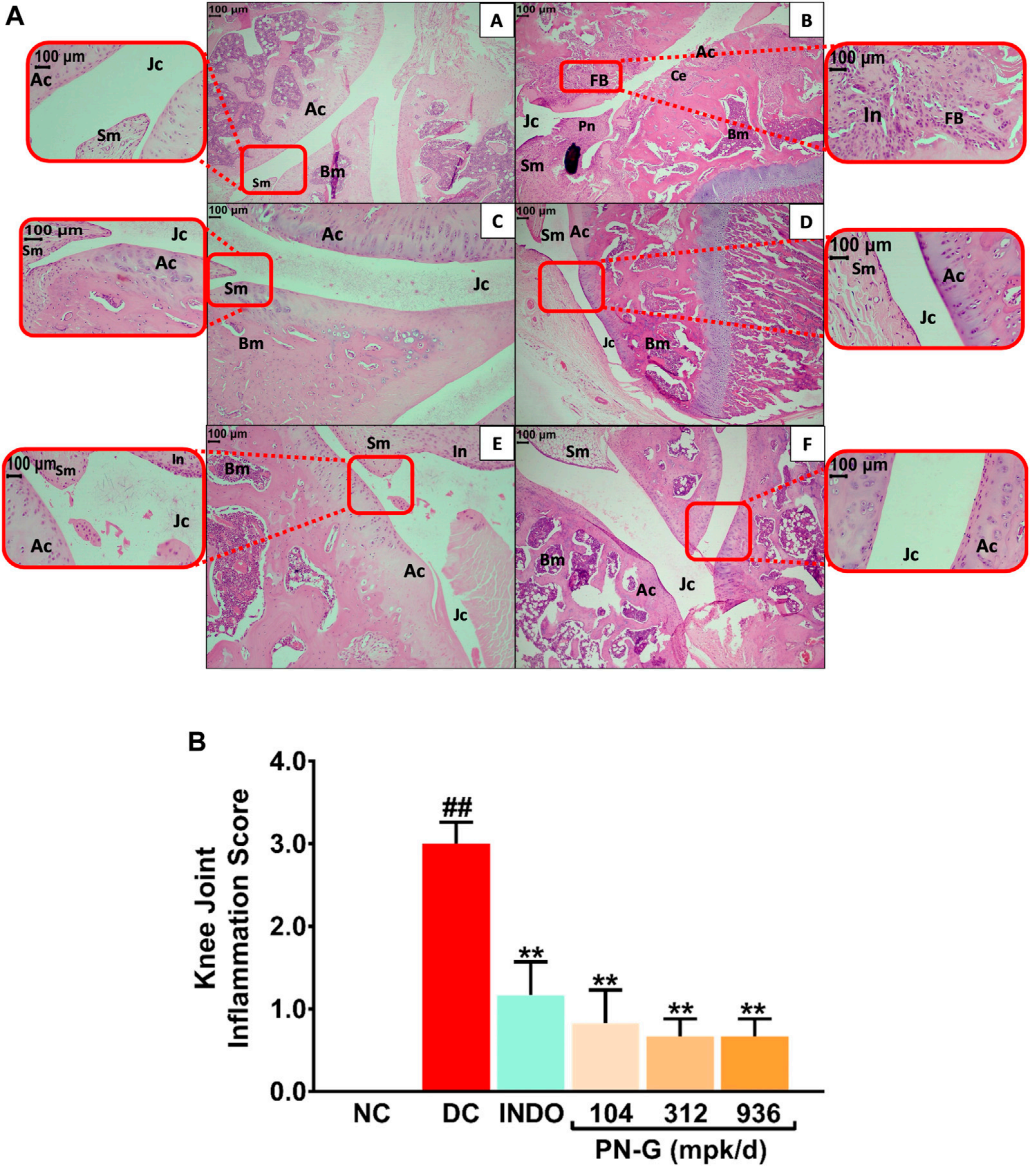
PN-G prevented the inflammation in osteoarthritic joint tissues. **(A)** Representative micrographs of H-E stained right knee joint sections, at ×10 and ×40 magnifications, from NC **(A)**, DC **(B)**, INDO **(C)**, PN-G 104 **(D)**, PN-G 312 **(E)** and PN-G 936 **(F)** groups depicting various histopathological features, like, normal articular cartilage (Ac), bone marrow cells (Bm), synovial membrane (Sm), joint cavity (Jc), cartilage erosion (Ce), superficial fibrillation of the articular cartilage (FB), inflammation (In) and pannus formation (Pn). **(B)** Bar graph depicting the knee joint inflammation score. Data is presented as mean ± SEM (*n* = 6 animals per group) and was statistically analyzed using one-way ANOVA followed by Dunnett’s multiple comparison test. ^##^ depicts significant difference with respect to NC (*p* < 0.01) whereas ** shows a statistically significant effect when compared to DC (*p* < 0.01).

Innate immunity plays an important role in osteoarthritic pathogenesis, and inflammation is one of the primary manifestations of innate immunogenic responses (Sokolove and Lepus, 2013; Xiao, 2017). Inflammation has been identified as a major risk factor for osteoarthritis. In fact, chronic inflammation can be a prelude to onset of osteoarthritis (Sokolove and Lepus, 2013). Synovial inflammation facilitates cartilage damage, precedes characteristic structural changes and consequently leads to osteoarthritis (Goldring and Otero, 2011; Sokolove and Lepus, 2013). Therefore, targeting inflammation could be a potential strategy to manage osteoarthritis. Given the observed *in vitro* anti-inflammatory property of PN-G, it becomes apposite to check its anti-inflammatory effect *in vivo*. Therefore, sections of osteoarthritic knee joints of rats were H-E stained and histopathologically scored (Figure 6). Normal articular cartilage (Ac), synovial membrane (Sm), bone marrow cells (Bm) and joint cavity (Jc) were visible in histological knee joint sections from NC group (Figure 6AA). MIA-induced osteoarthritis in the rats of DC group was histologically confirmed through cartilage erosion (Ce), inflammation (In), pannus formation (Pn) and superficial fibrillation of the articular cartilage (FB). Synovial hyperplasia leading to reduced joint cavity (Jc) is distinctly visible in the osteoarthritic joint section (Figure 6AB). In indomethacin treated section, the synovial hyperplasia and enlargement of synovial lining was completely absent, and so were articular cartilage fibrillation (FM) and pannuses, thereby indicating osteoarthritic amelioration (Figure 6AC). Likewise, PN-G treatments also led to the absence of these characteristic histological features of osteoarthritis (Figure 6AD–F). Osteoarthritis in the DC group was reflected in the relatively high overall severity score (3.00 ± 0.26) of the microscopic arthritic changes, like, synovial lining enlargement, synovial hyperplasia, synovial vascularity, infiltration of inflammatory cells, pannus formation, cartilage and bone erosion. This score was reduced by more than half with treatments with indomethacin or PN-G. In fact, PN-G treatments appeared to be more effective in abolishing histological features of osteoarthritis (Figure 6B).

**FIGURE 7:**
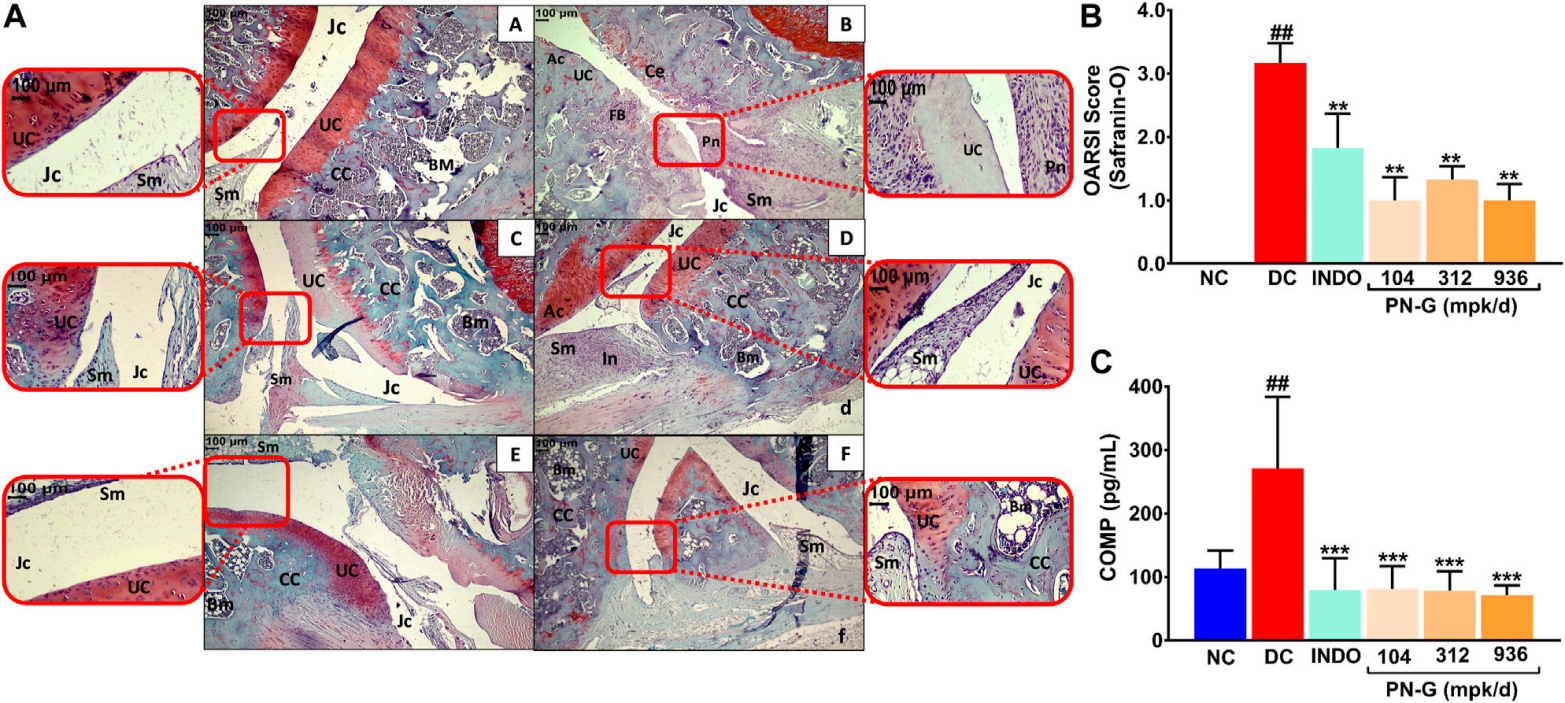
PN-G protects against cartilage degeneration, a characteristic anomaly representative of osteoarthritis. **(A)** Representative micrographs of H-E and Safranin O stained right knee joint sections, at ×10 and ×40 magnifications, from NC **(A)**, DC **(B)**, INDO **(C)**, PN-G 104 **(D)**, PN-G 312 **(E)** and PN-G 936 **(F)** groups depicting various histopathological features, like, uncalcified cartilage (UC), calcified cartilage (CC), synovial membrane (Sm), joint cavity (Jc) and bone marrow (BM), cartilage erosion (Ce), superficial fibrillation of the articular cartilage (FB), inflammation (In) and pannus formation (Pn). **(B)** Bar graph depicting the OARSI score for extent of cartilage degeneration. **(C)** Graphical representation of the effect of PN-G treatment on the serum osteoarthritic biomarker, Cartilage Oligomeric Matrix Protein (COMP). All data presented as mean ± SEM (*n* = 6 animals per group) and was statistically analyzed using one-way ANOVA followed by Dunnett’s multiple comparison test. ^##^ depicts significant difference with respect to NC (*p* < 0.01) whereas ** and *** show a statistically significant effect when compared to DC (**p < 0.01 and ***p < 0.001).

Cartilage degeneration is a hallmark of osteoarthritic pathogenesis (Pap and Korb-Pap, 2015). Therefore, as a part of its anti-osteoarthritic effects, the ability of PN-G to facilitate articular cartilage regeneration was assessed through H & E and Safranin O staining of knee joint sections, followed by OARSI scoring. Normal uncalcified cartilage (UC, red zone), calcified cartilage (CC), synovial membrane (Sm), joint cavity (Jc) and bone marrow (BM) were visible in histological sections from NC group (Figure 7AA). Characteristic cartilage erosion (Ce), superficial fibrillation of the articular cartilage (FB), inflammation (In), and pannus formation (Pn) were observed in the section from DC group. The section had significantly reduced uncalcified cartilage (UC), evident from absence of Safranin O stained red zone at the articular interfaces (Figure 7AB). With indomethacin and PN-G treatments, the Safranin O stainable uncalcified cartilage re-appeared, besides, abolishment of the characteristic osteoarthritic features (Figure 7AC–F). The cartilage regenerative effects of indomethacin and PN-G were comparable. With an OARSI score of 3.17 ± 0.31, DC group showed significant cartilage degeneration. The sizeable reductions in this score to half and below for indomethacin and PN-G treatments, respectively, indicates cartilage regeneration in these groups (Figure 7B). Cartilage Oligomeric Matrix Protein (COMP) is a biomarker of arthritis, including osteoarthritis, particularly, knee osteoarthritis (Tseng et al., 2009; Verma and Dalal, 2013; Bi, 2018). Therefore, COMP levels were measured and as expected was found to be increased in DC group, whereas, treatments with either indomethacin or PN-G significantly reduced its levels (Figure 7C). Taken together, these observations demonstrated that PN-G could modulate inflammatory pathways to manage osteoarthritic pathogenesis, which could be clinically monitored through the established osteoarthritic biomarker, COMP.

## 3 Discussion

Osteoarthritis has a multifactorial etiology, with inflammation and oxidative stress going hand-in-hand for promoting disease progression. Inflammatory mediators, like, IL-6, IL-1β, among several other factors play a crucial role in the pathogenesis of osteoarthritis (Sokolove and Lepus, 2013). Likewise, oxidative stress imparts strong facilitative impact on the osteoarthritic pathophysiology through chondrocyte senescence and synovial inflammation (Lepetsos and Papavassiliou, 2016), thus, being recognized as a potential therapeutic target (Poulet and Beier, 2016). The observed anti-osteoarthritic effect of PN-G is also multifactorial. The rich phyto-constitution of PN-G is likely to target several etiological factors of osteoarthritis. The collection of marker phytochemicals, including ellagic acid, guggulsterone E, guggulsterone Z, 5-(hydroxymethyl) furfural, corilagin, cinnamic acid, ferulic acid, gallic acid and protocatechuic acid, detected in PN-G accounts for its *in vitro* anti-inflammatory and *in vivo* nociception modulatory effects (Liao et al., 2012; Shah et al., 2012; Jin et al., 2013; Moreira et al., 2013; Trevisan et al., 2014; Naghizadeh et al., 2016; Zhang et al., 2016; BenSaad et al., 2017; Priebe et al., 2018; Zhang et al., 2018; Kong et al., 2019). MIA-induced osteoarthritis in rats leads to inflammation-mediated articular cartilage degeneration (McCoy, 2015). The inhibition and subsequent reversal of this cartilage degeneration observed in the PN-G treated animals implicated that its *in vitro* anti-inflammatory property is relatable *in vivo* also. In fact, a recent study has reported that ellagic acid inhibited osteoarthritic progress in the mouse surgical destabilization of the medial meniscus model (DMM), through, downregulation of inflammatory modulators, like, IL-6 and IL-1β and suppression of NF-κB signaling. This, in turn, inhibited IL-1β-induced cyclooxygenase-2 (COX-2) and inducible nitric oxide synthase (iNOS) expressions, thereby, indicating that ellagic acid could also modulate the oxidative stress (Lin et al., 2020), implicated in osteoarthritis (Sokolove and Lepus, 2013). Ferulic acid also has modulatory effects on IL-6 and IL-1β, although, tested in the context of airway inflammation (Zhang et al., 2018). Nevertheless, this information is relevant in accounting for the observed anti-inflammatory effect of PN-G. Like ellagic acid, 5-(hydroxymethyl) furfural (Kong et al., 2019) and cinnamic acid (Liao et al., 2012) could downregulate inflammatory modulators, such as COX-2 and iNOS, in *in vitro* inflammatory model and *in vivo* carrageenan-induced paw edema model, respectively. Similarly, the anti-osteoarthritic effect of protocatechuic acid has been attributed to its anti-oxidant property (Lende et al., 2011). In supporting literature, the anti-inflammatory and anti-oxidant properties of some of the PN-G marker phytocompounds, such as, ferulic acid, 5-(hydroxymethyl) furfural and cinnamic acid have been validated in the context of general inflammation. Nevertheless, such observations can be co-related to the current context to logically deduce that inflammatory processes responsible for osteoarthritis are being modulated by PN-G because of these phytocompounds.

Co-involvement of inflammation mediators, like, IL-6, IL-1β and NF-κB in osteoarthritic pathogenesis is a well-established fact widely reported in the literature (Sokolove and Lepus, 2013). The effect of PN-G on the changes in the levels of pro-inflammatory cytokines, IL-6 and IL-1β were representatively monitored. Nonetheless, a speculative discussion on putative effect of PN-G, given its NF-κB-modulatory phytoconstituents, is warranted. NF-kB activation, leading to cartilage degeneration (Choi et al., 2019), plays an important role in the osteoarthritis pathogenesis (Rigoglou and Papavassiliou, 2013), thus, being recognized as a potential therapeutic target (Roman-Blas and Jimenez, 2006). Guggulsterone Z has been found to inhibit NF-κB activation, although, in a different context of lung cancer and leukemia (Shishodia and Aggarwal, 2004). Nevertheless, NF-κB signaling is a shared pathway in pathogenesis of several diseases, implicating that guggulsterone Z present in PN-G is likely to impart similar benefits in osteoarthritis as well. Considering the common NF-κB-modulatory property, reported in four out of its nine phytoconstituents, namely, ellagic acid, guggulsterone, corilagin and cinnamic acid, PN-G is most likely to affect NF-κB signaling as a part of its anti-osteoarthritic effect. Transient Receptor Potential Ankyrin 1 (TRPA1) is implicated in osteoarthritis. TRPA1 upregulates IL-6 levels in osteoarthritic chondrocyte models (Nummenmaa et al., 2021). TRPA1 deficient mice had reduced inflammation and joint pain with MIA injection (Moilanen et al., 2015). Gallic acid can alleviate TRPA1 mediated inflammatory nociception (Trevisan et al., 2014), thereby indicating that PN-G might be effective against TRPA1-mediated osteoarthritic pathogenesis.

Osteoarthritic pain has two components: the osteoarthritic component arising due to cartilage degeneration and the neuropathic component resulting from the neural damage due to disease progression. Thus, inflammation preludes both the pain components, but, the inflamed tissues involved are different. However, in the current model, these components are not distinguishable. Chondrocytic death due to MIA-induced osteoarthritis leads to damage-associated molecular pattern (DAMP)-mediated inflammatory response (Eitner et al., 2017; De Sousa Valente, 2019). This, in turn, leads to articular cartilage degeneration generating the osteoarthritic pain phenotype, that is sensitive to NSAIDs (Eitner et al., 2017). The articular mechanosensitive ion channels (MSICs) get sensitized during osteoarthritis leading to allodynia (He et al., 2017) contributing to the neuropathic pain component of osteoarthritis. Reduction in IL-6 and IL-1β in the current study suggests that observed pain alleviation by PN-G in MIA-induced osteoarthritic rats is possibly due to its anti-inflammatory property. Given that both allodynia (representing neuropathic pain) and hyperalgesia (arising from cartilage degeneration) were alleviated with PN-G treatment, it is possible that this poly-herbo-mineral pain-relieving formulation is effective against both the components of osteoarthritic pain. However, a better understanding regarding this will require further detailed studies. Nevertheless, while most common osteoarthritic treatments aim at symptomatic alleviation, PN-G targets inflammation, the pathophysiological cause of osteoarthritis. The differentiated mode-of-action of PN-G holds promise for being rather effective osteoarthritic treatment option, with a potential of significantly reducing disease persistence, in a longer run.

The current study was designed to establish the anti-osteoarthritic potential of a classical Ayurvedic poly-herbo-mineral formation using the well-established, MIA-induced osteoarthritic rat model. The anti-osteoarthritic effects of PN-G were confirmed radiologically, histopathologically and biochemically. It was observed that PN-G was equally effective against osteoarthritis as the standard drug, indomethacin, used as positive control in the study. PN-G effectively restored knee joint morphology, alleviated osteoarthritic pain phenotypes and reversed cartilage degeneration. In conclusion, this study has succinctly demonstrated that PN-G is capable of relieving the clinical symptoms of osteoarthritis, which is measurable through the established osteoarthritic serum biomarker, Cartilage Oligomeric Matrix Protein (COMP).

## 4 Materials and Methods

### 4.1 Chemicals and Reagents

Peedanil Gold (PN-G) (batch #APGT20005) was sourced from Divya Pharmacy, Haridwar, Uttarakhand, India. Cell culture media, RPMI-1640 (cat #31800) was procured from Gibco (Amarillo, TX, United States). Fetal bovine serum (FBS) and Alamar blue (cat #TC235-1G) were purchased from HiMedia (Mumbai, India). Penicillin–streptomycin (100 U/ml) (cat #P4333), lipopolysaccharide (LPS) (cat #L2630-10MG), Monosodium iodoacetate (MIA) (cat #I2512-25G), and indomethacin (INDO) (cat #I7378-10G) were purchased from Sigma Aldrich (St. Louis, MO, United States). Human interleukin-6 (IL-6) (cat #555220) and human interleukin-1 beta (IL-1β) (cat #557953) ELISA kits were purchased from BD Biosciences (Franklin Lakes, NJ, United States). Rat Cartilage Oligomeric Matrix Protein (COMP) (cat #CSB-E13833r) ELISA kits were purchased from Cusabio Technologies, LLC (Houston, TX, United States). Phorbol 12-myristate 13-acetate (PMA) (cat #K63916) was from Alfa Aesar (Haverhill, MA, United States). Human monocytic THP-1 cell line (RRID: CVCL_0006) was procured from the ATCC recognized repository at National Centre for Cell Science, Pune, Maharashtra, India. 8-week-old male Sprague Dawley (SD) rats (230–270 g) were purchased from Charles River Laboratory licensed supplier, Hylasco Biotechnology Pvt. Ltd., Hyderabad, India. Standard pellet diets for these animals were procured from Purina Lab Diet (St. Louis, MO, United States).

### 4.2 Compositional Analysis

The marker phytocompounds of Peedanil Gold (PN-G) were detected through Ultra High Performance Liquid Chromatography (UHPLC) using a Photodiode Array (PDA) detector, and were identified and quantified against known reference standards, available commercially. UHPLC-PDA analysis was performed on Prominence-XR UHPLC system (Shimadzu, Japan) equipped with Quaternary pump (NexeraXR LC-20AD XR), PDA detector (SPD-M20 A), auto-sampler (Nexera XR SIL-20 AC XR), degassing unit (DGU-20A 5R), and column oven (CTO-10 AS VP). Separation of marker phytocompounds was carried out on a Shodex C18-4E (5 μm, 4.6*250 mm) (Shodex, Japan) column at 35.0°C. An injected volume of 10 µl PN-G was subjected to binary gradient elution, at a flow rate of 1.0 ml/min. The two solvents used for the analysis consisted of water containing 0.1% orthophosphoric acid; adjust pH 2.5 with diethyl amine (solvent A) and Acetonitrile (solvent B). Gradient programming of the solvent system was initially at 5–10% B for 0–10 min, 10–35% B from 10 to 30 min, 35–50% B from 30 to 40 min, 50–75% B from 40 to 50 min, 75% B from 50 to 55 min, 75–90% B from 55 to 60 min, 90% B from 60 to 65 min, 90 to 5% B from 65 to 66 min and 5% B from 66 to 70 min. The chromatographic detection of all the analytes was performed using a PDA detector at 250 nm. PN-G test solution was prepared by suspending 0.5 gm of powdered PN-G tablet in 10 ml water: methanol (20:80) with sonication for 30 min. Subsequently the suspension was centrifuged at 10,000 rpm for 5 min and filtered through 0.45 µm nylon filter before conducting the UHPLC-PDA analysis. Commercially procured gallic acid (purity: 97.3%, Sigma Aldrich, St. Louis, MO, United States), 5-hydroxymethyl-2-furaldehyde (5-HMF) (purity: 97.3%, TCI Chemicals (India) Pvt. Ltd. Gurugram, Haryana, India), protocatechuic acid (purity: 99.5%, Natural remedies, Bengaluru, Karnataka, India), corilagin (purity: 98.0%, Chem Faces, Wuhan, Hubei, China), ellagic acid (purity: 99.6%, Sigma Aldrich, St. Louis, MO, United States), ferulic acid (purity: 99.0%, Natural Remedies, Bengaluru, Karnataka, India), cinnamic acid (purity: 99.7%, SRL Chemicals, Gurugram, Haryana, India), guggulsterone E (purity: 98.1%, Natural remedies, Bengaluru, Karnataka, India) and guggulsterone Z (purity: 97.6%, Natural remedies, Bengaluru, Karnataka, India) were individually dissolved in methanol to prepare 1,000 ppm standard solutions. Working standard was prepared by mixing 0.05 ml each of 1,000 ppm standard stock solution and diluting it to 1 ml to prepare 50 ppm of working standard mix. Quantification of different phytochemicals was performed by using individual reference standard mixed together in a solution. Validation of analytical method for all marker compounds was performed as per international guidelines ICH-Q2 (R1) (https://www.ema.europa.eu/en/ich-q2-r1-validation-analytical-procedures-text-methodology) and United State Pharmacopeia (USP <1225>) (http://www.uspbpep.com/usp29/v29240/usp29nf24s0_c1225.html). The determined limits of detection (LOD) and quantification (LOQ) for gallic acid, 5-HMF, protocatechuic acid, corilagin and ellagic acid were 0.1 and 0.3 μg/g, respectively, those of cinnamic acid, guggulsterone E and guggulsterone Z are 0.3 and 1.0 μg/g, whereas, LOD and LOQ for ferulic acid were found to be 1.0 and 2.0 μg/g. The relative standard deviation (RSD) for LOD and LOQ across replicate injections were less than 33 and 10%, respectively. Linearity was observed over a range of 0.3–100 μg/g with a coefficient of correlation (*r* ^2^) of 0.99.

### 4.3 *In Vitro* Cytosafety and Anti-Inflammatory Assays

Cytosafety and anti-inflammatory effect of PN-G was evaluated in THP-1 cells, as described earlier (Balkrishna et al., 2020). Cells, seeded at a density of 5 × 10^4^ cells/well, were differentiated into macrophages with 20 ng/ml with PMA for 16 h before treating with different concentrations (1, 3, 10 and 30 μg/ml) of PN-G for another 24 h. Subsequently, inflammation was induced in these cells with LPS (500 ng/ml) and further co-treated with the above mentioned concentrations of PN-G for the entire duration of inflammation induction of 24 h. Levels of secreted IL-6 and IL-1β in the cell supernatants were measured using ELISA kits according to manufacturer’s protocol. Absorbance was recorded at 450 nm using the Envision microplate reader (Perkin Elmer, United States). Viability of the same cells were determined through Alamar blue staining. After washing the treated cells with PBS, 100 µl Alamar blue (15 μg/ml) was added to each well, and incubated for 3 h at 37°C. Subsequently, fluorescence was measured using Envision microplate reader (Perkin Elmer, United States) at 530 (excitation) and 590 (emission) nm wavelengths.

### 4.4 *In Vivo* Anti-Osteoarthritic Study

#### 4.4.1 Development of Osteoarthritic Model and Treatment Plan

Animal study was conducted according to a protocol, approved by IEAC (PRIAS/LAF/IAEC-084) following the guidelines of the Committee for the Purpose of Control and Supervision of Experiments on Animals (CPCSEA), Govt. of India. After a 7-day quarantine, 8-week old male Sprague Dawley rats weighing 230–270 g were randomized and osteoarthritic model was established according to earlier reports (Kobayashi et al., 2003; Izumi et al., 2012; Udo et al., 2016), and as shown in Figure 3. Briefly, the osteoarthritic model was induced through a one-time intra-articular injection of Monosodium iodoacetate (MIA) at a concentration of 3 mg/50 µl of normal saline and was allowed to develop over a period of 7 days. Thereafter, the animals were divided into six groups, which received their designated treatments as detailed in Figure 3. Clinical scoring and pain assessments were conducted during the treatment regime, radiography was done at the termination of the experiment, before sacrifice, and knee joint histological analysis and serum Cartilage Oligomeric Matrix Protein (COMP) determination were carried out post-sacrifice, by using ELISA kits as per manufacturer instructions.

#### 4.4.2 Clinical Scoring for Osteoarthritis

For clinical scoring of MIA-induced osteoarthritis and its abrogation due to treatments, the rats were inspected for swellings in knee joints and limping on days 0, 7, 11 and 14. The animals were classified as no change, mild, and severe on the basis of severity scale, as defined elsewhere (Choi et al., 2002). Clinical scoring was done by a researcher who was blinded to the treatment conditions.

#### 4.4.3 Kellgren and Lawrence Scoring

The extent of osteoarthritic progress were analyzed through the classical Kellgren and Lawrence (K & L) scoring of the knee joint radiograms (Kellgren and Lawrence, 1957). Each radiogram was assigned a grade from 0 to 4, correlating to increasing severity of osteoarthritis, with grade 0 signifying no disease and grade 4 signifying severe osteoarthritis. A K & L score of 1 is defined as doubtful joint space narrowing (JSN) and possible osteophytic lipping. A K & L score of 2, denotes the presence of definite osteophytes and possible JSN. Further disease progression is graded with a K & L score of 3, characterized by multiple osteophytes, definite JSN, sclerosis, possible bony deformity. K & L grade 4 is assigned when there is presence of large osteophytes, marked JSN, severe sclerosis and definitive bony deformity (Belkhodja et al., 2017; Abdel Jaleel et al., 2020).

#### 4.4.4 Pain Assessments

Mechanical hyperalgesia measurement was done on days 7, 11, 14, 18, 21 and 25 through Pressure Application Mechanics (PAM), as reported earlier (Fernihough et al., 2004). The pain response, to the mechanical stimulus of limb pulling as limb withdrawal threshold (LWT; g), was recorded as the pressure which incited the animal to withdraw its limb. A pressure of 300 g/s for 5 s was set as the maximum limit to avoid tissue damage. Readouts were recorded by a researcher blinded to the treatment conditions.

Tactile allodynia measurement was done on days 8, 12, 15, 19, 22 and 26, through the classical Von Frey test (Electronic Von Frey, UgoBasile, VA, Italy; Model: 38450-001), as reported earlier (Fernihough et al., 2004). Briefly, the animals were habituated, for 45 min for 3 consecutive days and acclimatized for 10 min, in transparent Perspex cubicles with elevated wire mesh bottom prior to the test. Pressure applied to the right hind paw of each animal with Von Frey filament (0.5 mm diameter) was slowly increased until, responses like, lifting, licking or shaking of the paw were observed and noted as paw withdrawal threshold (PWT; g). A pressure of 10 g/s for 5 s was set as the maximum limit to avoid tissue damage. Researcher recording the readouts was blinded to the treatment conditions.

#### 4.4.5 Histopathology

Sections of knee joints were subjected to histopathological analysis for evaluating the osteoarthritic pathology at the tissue level. The right limb was harvested from humanely euthanized rats, fixed in 10% buffered neutral formalin, decalcified in 10% formic acid for 4 days, embedded in paraffin wax, sliced in solid sections of 3- to 5-μm thickness, and stained with hematoxylin and eosin (H-E) for general evaluation and Safranin-O stain for specific assessment of cartilage damage. Blinded examination of histological slides was performed by an external veterinary pathologist to minimize bias. The severity of microscopic arthritic changes (enlargement in synovial lining cell layer, synovial hyperplasia, synovial vascularity, infiltration of inflammatory cells, pannus formation, cartilage erosion, and bone erosion) was evaluated in H-E-stained slides using the following grades: 0 = no abnormality detected; 1 = minimal (<1%); 2 = mild (1%–25%); 3 = moderate (26%–50%); 4 = marked (51%–75%); 5 = severe (76%–100%) (Gerwin et al., 2010). The severity of OA was evaluated in the medial compartment of the knee using the Osteoarthritis Research Society International (OARSI) scoring system after staining with H-E and Safranin-O (Gerwin et al., 2010). The cartilage degeneration was scored using a scoring system that measured on a scale of 0–5 (0 = no degeneration, 1 = mild degeneration in the surface, 2 = slightly extended degeneration in the upper center, 3 = moderate degeneration in the median area, 4 = extended deep degeneration, and 5 = severe degeneration. Images of the histological slides (H-E and Safranin-O) were captured at low (×10) and high (×40) magnifications using Nikon E100 (Eclipse) microscope camera and processed by GT 5.0 image analysis software.

### 4.5 Data Representation and Statistical Analysis

Data are expressed as mean ± standard error of the mean (SEM) for each group using GraphPad Prism version 7.0 software (San Diego, CA, United States). Statistical analysis of all quantitative data was carried out using in-built “analysis” option of GraphPad Prism. One-way analysis of variance (one-way ANOVA) was used to measure the statistical significance of the observed difference between more than two groups. *p*-values < 0.05 were considered statistically significant.

## Data Availability

The original contributions presented in the study are included in the article/Supplementary Material, further inquiries can be directed to the corresponding author.

## Footnotes

1
https://www.cdc.gov/arthritis/basics/osteoarthritis.htm Osteoarthritis (OA). Centers Dis. Control Prev., 11–13. Available at: https://www.cdc.gov/arthritis/basics/osteoarthritis.htm.

2
https://oarsi.org/sites/default/files/docs/2016/oarsi_white_paper_oa_serious_disease_121416_1.pdf (2016). Osteoarthritis: A Serious Disease, Submitted to the U.S. Food and Drug Administration. Osteoarthr. Res. Soc. Int., 1–102. Available at: https://oarsi.org/sites/default/files/docs/2016/oarsi_white_paper_oa_serious_disease_121416_1.pdf [Accessed November 12, 2021].

## References

[B1] Abdel Jaleel G. A. Saleh D. O. Al-Awdan S. W. Hassan A. Asaad G. F. (2020). Impact of Type III Collagen on Monosodium Iodoacetate-Induced Osteoarthritis in Rats. Heliyon 6, e04083. 10.1016/j.heliyon.2020.e04083 32548322 PMC7284073

[B2] Balkrishna A. Sakat S. S. Karumuri S. Singh H. Tomer M. Kumar A. (2020). Herbal Decoction Divya-Peedantak-Kwath Alleviates Allodynia and Hyperalgesia in Mice Model of Chemotherapy-Induced Peripheral Neuropathy via Modulation in Cytokine Response. Front. Pharmacol. 11, 1–19. 10.3389/fphar.2020.566490 33324205 PMC7723448

[B3] Belkhodja H. Meddah B. Meddah TirTouil A. Slimani K. Tou A. (2017). Radiographic and Histopathologic Analysis on Osteoarthritis Rat Model Treated with Essential Oils of Rosmarinus Officinalis and Populus Alba. Pharm. Sci. 23, 12–17. 10.15171/PS.2017.03

[B4] BenSaad L. A. Kim K. H. Quah C. C. Kim W. R. Shahimi M. (2017). Anti-inflammatory Potential of Ellagic Acid, Gallic Acid and Punicalagin A&B Isolated from Punica Granatum. BMC Complement. Altern. Med. 17, 47–10. 10.1186/s12906-017-1555-0 28088220 PMC5237561

[B5] Coxib and traditional NSAID Trialists (CNT) Bhala N. Emberson J. Merhi A. Abramson S. Arber N. Baron J. A. (2013). Vascular and Upper Gastrointestinal Effects of Non-steroidal Anti-inflammatory Drugs: Meta-Analyses of Individual Participant Data from Randomised Trials. Lancet 382, 769–779. 10.1016/S0140-6736(13)60900-9 23726390 PMC3778977

[B6] Bi X. (2018). Correlation of Serum Cartilage Oligomeric Matrix Protein with Knee Osteoarthritis Diagnosis: A Meta-Analysis. J. Orthop. Surg. Res. 13, 262–268. 10.1186/s13018-018-0959-y 30340615 PMC6195708

[B7] Choi J. H. Choi J. H. Kim D. Y. Yoon J. H. Youn H. Y. Yi J. B. (2002). Effects of SKI 306X, a New Herbal Agent, on Proteoglycan Degradation in Cartilage Explant Culture and Collagenase-Induced Rabbit Osteoarthritis Model. Osteoarthritis Cartilage 10, 471–478. 10.1053/joca.2002.0526 12056850

[B8] Choi M. C. Jo J. Park J. Kang H. K. Park Y. (2019). NF-κB Signaling Pathways in Osteoarthritic Cartilage Destruction. Cells 8, 734. 10.3390/cells8070734 PMC6678954 31319599

[B9] Chow Y. Y. Chin K.-Y. (2020). The Role of Inflammation in the Pathogenesis of Osteoarthritis. Mediators Inflamm. 2020, 1–19. 10.1155/2020/8293921 PMC7072120 32189997

[B10] De Sousa Valente J. (2019). The Pharmacology of Pain Associated with the Monoiodoacetate Model of Osteoarthritis. Front. Pharmacol. 10, 1–8. 10.3389/fphar.2019.00974 31619987 PMC6759799

[B11] Eitner A. Hofmann G. O. Schaible H.-G. (2017). Mechanisms of Osteoarthritic Pain. Studies in Humans and Experimental Models. Front. Mol. Neurosci. 10, 1–22. 10.3389/fnmol.2017.00349 29163027 PMC5675866

[B12] Fernihough J. Gentry C. Malcangio M. Fox A. Rediske J. Pellas T. (2004). Pain Related Behaviour in Two Models of Osteoarthritis in the Rat Knee. Pain 112, 83–93. 10.1016/j.pain.2004.08.004 15494188

[B13] Gerwin N. Bendele A. M. Glasson S. Carlson C. S. (2010). The OARSI Histopathology Initiative - Recommendations for Histological Assessments of Osteoarthritis in the Rat. Osteoarthritis Cartilage 18 Suppl 3, S24–S34. 10.1016/j.joca.2010.05.030 20864021

[B14] Goldring M. B. Otero M. (2011). Inflammation in Osteoarthritis. Curr. Opin. Rheumatol. 23, 471–478. 10.1097/BOR.0b013e328349c2b1 21788902 PMC3937875

[B15] Gregori D. Giacovelli G. Minto C. Barbetta B. Gualtieri F. Azzolina D. (2018). Association of Pharmacological Treatments with Long-Term Pain Control in Patients with Knee Osteoarthritis: A Systematic Review and Meta-Analysis. JAMA 320, 2564–2579. 10.1001/jama.2018.19319 30575881 PMC6583519

[B17] He B. H. Christin M. Mouchbahani-Constance S. Davidova A. Sharif-Naeini R. (2017). Mechanosensitive Ion Channels in Articular Nociceptors Drive Mechanical Allodynia in Osteoarthritis. Osteoarthritis Cartilage 25, 2091–2099. 10.1016/j.joca.2017.08.012 28882752

[B18] Izumi M. Ikeuchi M. Ji Q. Tani T. (2012). Local ASIC3 Modulates Pain and Disease Progression in a Rat Model of Osteoarthritis. J. Biomed. Sci. 19, 77. 10.1186/1423-0127-19-77 22909215 PMC3520115

[B20] Jin F. Cheng D. Tao J. Y. Zhang S. L. Pang R. Guo Y. J. (2013). Anti-inflammatory and Anti-oxidative Effects of Corilagin in a Rat Model of Acute Cholestasis. BMC Gastroenterol. 13, 79. 10.1186/1471-230X-13-79 23641818 PMC3655894

[B21] Jin Y. Smith C. Monteith D. Brown R. Camporeale A. McNearney T. A. (2018). CGRP Blockade by Galcanezumab Was Not Associated with Reductions in Signs and Symptoms of Knee Osteoarthritis in a Randomized Clinical Trial. Osteoarthritis Cartilage 26, 1609–1618. 10.1016/j.joca.2018.08.019 30240937

[B22] Kellgren J. H. Lawrence J. S. (1957). Radiological Assessment of Osteo-Arthrosis. Ann. Rheum. Dis. 16, 494–502. 10.1136/ard.16.4.494 13498604 PMC1006995

[B23] Kloppenburg M. Berenbaum F. (2020). Osteoarthritis Year in Review 2019: Epidemiology and Therapy. Osteoarthritis Cartilage 28, 242–248. 10.1016/j.joca.2020.01.002 31945457

[B24] Kloppenburg M. Peterfy C. Haugen I. K. Kroon F. Chen S. Wang L. (2019). Phase IIa, Placebo-Controlled, Randomised Study of Lutikizumab, an Anti-interleukin-1α and Anti-interleukin-1β Dual Variable Domain Immunoglobulin, in Patients with Erosive Hand Osteoarthritis. Ann. Rheum. Dis. 78, 413–420. 10.1136/annrheumdis-2018-213336 30552176 PMC6390132

[B25] Kloppenburg M. Ramonda R. Bobacz K. Kwok W. Y. Elewaut D. Huizinga T. W. J. (2018). Etanercept in Patients with Inflammatory Hand Osteoarthritis (EHOA): A Multicentre, Randomised, Double-Blind, Placebo-Controlled Trial. Ann. Rheum. Dis. 77, 1757–1764. 10.1136/annrheumdis-2018-213202 30282670

[B26] Kobayashi K. Imaizumi R. Sumichika H. Tanaka H. Goda M. Fukunari A. (2003). Sodium Iodoacetate-Induced Experimental Osteoarthritis and Associated Pain Model in Rats. J. Vet. Med. Sci. 65, 1195–1199. 10.1292/jvms.65.1195 14665748

[B27] Kong F. Lee B. H. Wei K. (2019). 5-Hydroxymethylfurfural Mitigates Lipopolysaccharide-Stimulated Inflammation via Suppression of MAPK, NF-Κb and mTOR Activation in RAW 264.7 Cells. Molecules 24. 10.3390/molecules24020275 PMC6359491 30642099

[B29] Lende A. B. Kshirsagar A. D. Deshpande A. D. Muley M. M. Patil R. R. Bafna P. A. (2011). Anti-inflammatory and Analgesic Activity of Protocatechuic Acid in Rats and Mice. Inflammopharmacology 19, 255–263. 10.1007/s10787-011-0086-4 21748471

[B30] Leopoldino A. O. Machado G. C. Ferreira P. H. Pinheiro M. B. Day R. McLachlan A. J. (2019). Paracetamol versus Placebo for Knee and Hip Osteoarthritis. www.cochranelibrary.com.2, CD013273. 10.1002/14651858.CD013273 PMC6388567 30801133

[B31] Lepetsos P. Papavassiliou A. G. (2016). ROS/oxidative Stress Signaling in Osteoarthritis. Biochim. Biophys. Acta 1862, 576–591. 10.1016/j.bbadis.2016.01.003 26769361

[B32] Liao J.-C. Deng J.-S. Chiu C.-S. Hou W.-C. Huang S.-S. Shie P.-H. (2012). Anti-Inflammatory Activities ofCinnamomum cassiaConstituentsIn VitroandIn Vivo. Evidence-Based Complement. Altern. Med. 2012, 1–12. 10.1155/2012/429320

[B33] Lin Z. Lin C. Fu C. Lu H. Jin H. Chen Q. (2020). The Protective Effect of Ellagic Acid (EA) in Osteoarthritis: An *In Vitro* and *In Vivo* Study. Biomed. Pharmacother. 125, 109845. 10.1016/j.biopha.2020.109845 32058211

[B35] McAlindon T. E. LaValley M. P. Harvey W. F. Price L. L. Driban J. B. Zhang M. (2017). Effect of Intra-articular Triamcinolone vs Saline on Knee Cartilage Volume and Pain in Patients with Knee Osteoarthritis. Jama 317, 1967. 10.1001/jama.2017.5283 28510679 PMC5815012

[B36] McCoy A. M. (2015). Animal Models of Osteoarthritis: Comparisons and Key Considerations. Vet. Pathol. 52, 803–818. 10.1177/0300985815588611 26063173

[B37] Moilanen L. J. Hämäläinen M. Nummenmaa E. Ilmarinen P. Vuolteenaho K. Nieminen R. M. (2015). Monosodium Iodoacetate-Induced Inflammation and Joint Pain Are Reduced in TRPA1 Deficient Mice-Ppotential Role of TRPA1 in Osteoarthritis. Osteoarthritis Cartilage 23, 2017–2026. 10.1016/j.joca.2015.09.008 26521748

[B38] Moreira J. Klein-Júnior L. C. Cechinel Filho V. de Campos Buzzi F. (2013). Anti-hyperalgesic Activity of Corilagin, a Tannin Isolated from Phyllanthus Niruri L. (Euphorbiaceae). J. Ethnopharmacol. 146, 318–323. 10.1016/j.jep.2012.12.052 23333746

[B39] Muchedzi T. A. Roberts S. B. (2018). A Systematic Review of the Effects of Platelet Rich Plasma on Outcomes for Patients with Knee Osteoarthritis and Following Total Knee Arthroplasty. Surgeon 16, 250–258. 10.1016/j.surge.2017.08.004 28943099

[B40] Naghizadeh B. Mansouri M. T. Ghorbanzadeh B. (2016). Ellagic Acid Enhances the Antinociceptive Action of Carbamazepine in the Acetic Acid Writhing Test with Mice. Pharm. Biol. 54, 157–161. 10.3109/13880209.2015.1025288 25898222

[B41] Nummenmaa E. Hämäläinen M. Pemmari A. Moilanen L. J. Tuure L. Nieminen R. M. (2021). Transient Receptor Potential Ankyrin 1 (TRPA1) Is Involved in Upregulating Interleukin-6 Expression in Osteoarthritic Chondrocyte Models. Ijms 22, 87–14. 10.3390/ijms22010087 PMC7794684 33374841

[B42] Pap T. Korb-Pap A. (2015). Cartilage Damage in Osteoarthritis and Rheumatoid Arthritis-Ttwo Unequal Siblings. Nat. Rev. Rheumatol. 11, 606–615. 10.1038/nrrheum.2015.95 26195338

[B43] Poulet B. Beier F. (2016). Targeting Oxidative Stress to Reduce Osteoarthritis. Arthritis Res. Ther. 18, 32–16. 10.1186/s13075-015-0908-7 26818766 PMC4730642

[B44] Priebe A. Hunke M. Tonello R. Sonawane Y. Berta T. Natarajan A. (2018). Ferulic Acid Dimer as a Non-opioid Therapeutic for Acute Pain. J. Pain Res. 11, 1075–1085. 10.2147/JPR.S161161 29922083 PMC5997134

[B45] Rigoglou S. Papavassiliou A. G. (2013). The NF-Κb Signalling Pathway in Osteoarthritis. Int. J. Biochem. Cel Biol. 45, 2580–2584. 10.1016/j.biocel.2013.08.018 24004831

[B46] Roman-Blas J. A. Jimenez S. A. (2006). NF-kappaB as a Potential Therapeutic Target in Osteoarthritis and Rheumatoid Arthritis. Osteoarthritis Cartilage 14, 839–848. 10.1016/j.joca.2006.04.008 16730463

[B47] Shah R. Gulati V. Palombo E. A. (2012). Pharmacological Properties of Guggulsterones, the Major Active Components of Gum Guggul. Phytother Res. 26, 1594–1605. 10.1002/ptr.4647 22388973

[B48] Shishodia S. Aggarwal B. B. (2004). Guggulsterone Inhibits NF-Κb and IκBα Kinase Activation, Suppresses Expression of Anti-apoptotic Gene Products, and Enhances Apoptosis. J. Biol. Chem. 279, 47148–47158. 10.1074/jbc.M408093200 15322087

[B49] Sokolove J. Lepus C. M. (2013). Role of Inflammation in the Pathogenesis of Osteoarthritis: Latest Findings and Interpretations. Ther. Adv. Musculoskelet. Dis. 5, 77–94. 10.1177/1759720X12467868 23641259 PMC3638313

[B50] Stevens R. M. Ervin J. Nezzer J. Nieves Y. Guedes K. Burges R. (2019). Randomized, Double-Blind, Placebo-Controlled Trial of Intraarticular Trans-capsaicin for Pain Associated with Osteoarthritis of the Knee. Arthritis Rheumatol. 71, 1524–1533. 10.1002/art.40894 30888737 PMC6772016

[B51] Trevisan G. Rossato M. F. Tonello R. Hoffmeister C. Klafke J. Z. Rosa F. (2014). Gallic Acid Functions as a TRPA1 Antagonist with Relevant Antinociceptive and Antiedematogenic Effects in Mice. Naunyn. Schmiedebergs. Arch. Pharmacol. 387, 679–689. 10.1007/s00210-014-0978-0 24722818

[B52] Tseng S. Reddi A. H. Di Cesare P. E. (2009). Cartilage Oligomeric Matrix Protein (COMP): A Biomarker of Arthritis. Biomark. Insights 4, 33–44. 10.4137/bmi.s645 19652761 PMC2716683

[B53] Udo M. Muneta T. Tsuji K. Ozeki N. Nakagawa Y. Ohara T. (2016). Monoiodoacetic Acid Induces Arthritis and Synovitis in Rats in a Dose- and Time-dependent Manner: Proposed Model-specific Scoring Systems. Osteoarthritis Cartilage 24, 1284–1291. 10.1016/j.joca.2016.02.005 26915639

[B54] Verma P. Dalal K. (2013). Serum Cartilage Oligomeric Matrix Protein (COMP) in Knee Osteoarthritis: A Novel Diagnostic and Prognostic Biomarker. J. Orthop. Res. 31, 999–1006. 10.1002/jor.22324 23423905

[B55] Xiao T. S. (2017). Innate Immunity and Inflammation. Cell. Mol. Immunol. 14, 1–3. 10.1038/cmi.2016.45 27545072 PMC5214945

[B56] Xing D. Wang Q. Yang Z. Hou Y. Zhang W. Chen Y. (2018). Mesenchymal Stem Cells Injections for Knee Osteoarthritis: a Systematic Overview. Rheumatol. Int. 38, 1399–1411. 10.1007/s00296-017-3906-z 29273938

[B57] Zhang J. H. Shangguan Z. S. Chen C. Zhang H. J. Lin Y. (2016). Anti-inflammatory Effects of Guggulsterone on Murine Macrophage by Inhibiting LPS-Induced Inflammatory Cytokines in NF-Κb Signaling Pathway. Drug Des. Devel. Ther. 10, 1829–1835. 10.2147/DDDT.S104602 PMC4896467 27330276

[B58] Zhang S. Wang P. Zhao P. Wang D. Zhang Y. Wang J. (2018). Pretreatment of Ferulic Acid Attenuates Inflammation and Oxidative Stress in a Rat Model of Lipopolysaccharide-Induced Acute Respiratory Distress Syndrome. Int. J. Immunopathol. Pharmacol. 32, 394632017750518. 10.1177/0394632017750518 29350567 PMC5849244

